# Predictive brain signals best predict upcoming and not previous choices

**DOI:** 10.3389/fpsyg.2014.00406

**Published:** 2014-05-08

**Authors:** Chun S. Soon, Carsten Allefeld, Carsten Bogler, Jakob Heinzle, John-Dylan Haynes

**Affiliations:** ^1^Bernstein Center for Computational Neuroscience, Charité – Universitätsmedizin BerlinBerlin, Germany; ^2^Berlin Center for Advanced Neuroimaging, Charité – Universitätsmedizin BerlinBerlin, Germany; ^3^Berlin School of Mind and Brain, Humboldt-Universität zu BerlinBerlin, Germany; ^4^Neuroscience and Behavioral Disorders Program, Duke-NUS Graduate Medical SchoolSingapore; ^5^Department of Psychology, Technical University DresdenDresden, Germany; ^6^Translational Neuromodeling Unit, Institute for Biomedical Engineering, University of Zurich and ETH ZurichZurich, Switzerland

**Keywords:** mental state decoding, decision making, free choice, sequential dependency, random behavior

In a series of three human neuroimaging studies we recently identified prefrontal and parietal brain signals that predict a person's upcoming “free” choice up to several seconds before a person believes to be making up their mind (Soon et al., 2008, 2013; Bode et al., 2011). These findings were based on a combination of functional magnetic resonance imaging (fMRI) with multivariate pattern classification (e.g., Haynes and Rees, 2006) and extended similar previous work using electroencephalography measures where choice-predictive signals were also found, albeit across shorter time scales (e.g., Libet et al., 1983; Haggard and Eimer, 1999).

We took care to perform a number of sanity checks in order to rule out potential alternative explanations for our data (Soon et al., 2008), and more research will be needed to establish the nature and potential function of choice-predictive signals (Haynes, 2011). One of the questions we already originally addressed was whether our choice-predictive signals might reflect a spill-over from the previous trial, rather than being related to a process involved in shaping the upcoming choice. There were several aspects of our data that spoke against such an account. First, the classification accuracy increased with distance from the previous trial. Second, the classification of the time period of the next trial was not above chance, meaning that the regions we identified presumably did not carry predictive signals into the next trial. Third, we also used a simple assessment of randomness to check for dependencies between successive trials. We found that in our data sequence length roughly followed the shape of an exponential distribution, as would be expected for random data.

Lages et al. (2013) now raise the question whether this latter analysis, our assessment of randomness might not have been sufficient. We fully agree that the original approach was only an approximate assessment of randomness that can be improved in many ways. However, it was only one among a number of points that spoke against sequential dependencies. The proper assessment of randomness in the data from our choice experiments is tricky because the experimental data were acquired in separate runs, each of which only contained only around 10 choices. We recently published a reanalysis of the behavioral data from these studies (Allefeld et al., 2013) using more sensitive analyses based on single-subject data. A stochastic process analysis revealed that subjects' data was close to random with an entropy rate of around 0.95 bit/trial (where 1 bit/trial reflects perfectly random behavior). The redictability of a choice on trial N from the choice in the previous trial *N* − 1 was approximately 62–64%, based on a classifier that also exploited small base-rate differences between choices. This is in a similar range as between-trial predictability in other studies (e.g., Lopes, 1982), including those found by these authors (Lages and Jaworska, 2012).

Because the brain-based classification accuracies were on a similar scale to the trial-by-trial behavioral predictions one might be led to believe that the brain-based prediction reflects nothing other than the carry-over of information between trials (Lages et al., 2013). In the following we will explain why this inference is misguided and present data to show that our classification does not directly reflect signals from the previous trial. The “spillover model” postulates that the choice on the previous trial (*C*_*N* − 1_) has some form of temporal persistence in the brain that leads it to spill over into the next trial (*C*_*N*_), creating the false impression of a choice-predictive signal in trial *N* several seconds before the choice (Figure 1A). In this model the classifier simply picks up the spillover signal and is only predictive of the next trial because of the weak behavioral correlation. Please note, however, that because the predictive link between *C*_*N* − 1_ and *C*_*N*_ is weak (albeit existent), the brain-based classifier would need to be able to decode the brain signal related to *C*_*N* − 1_ with incredible precision in order to then be sufficiently predictable for *C*_*N*_, because the two are only weakly correlated.

**Figure 1:**
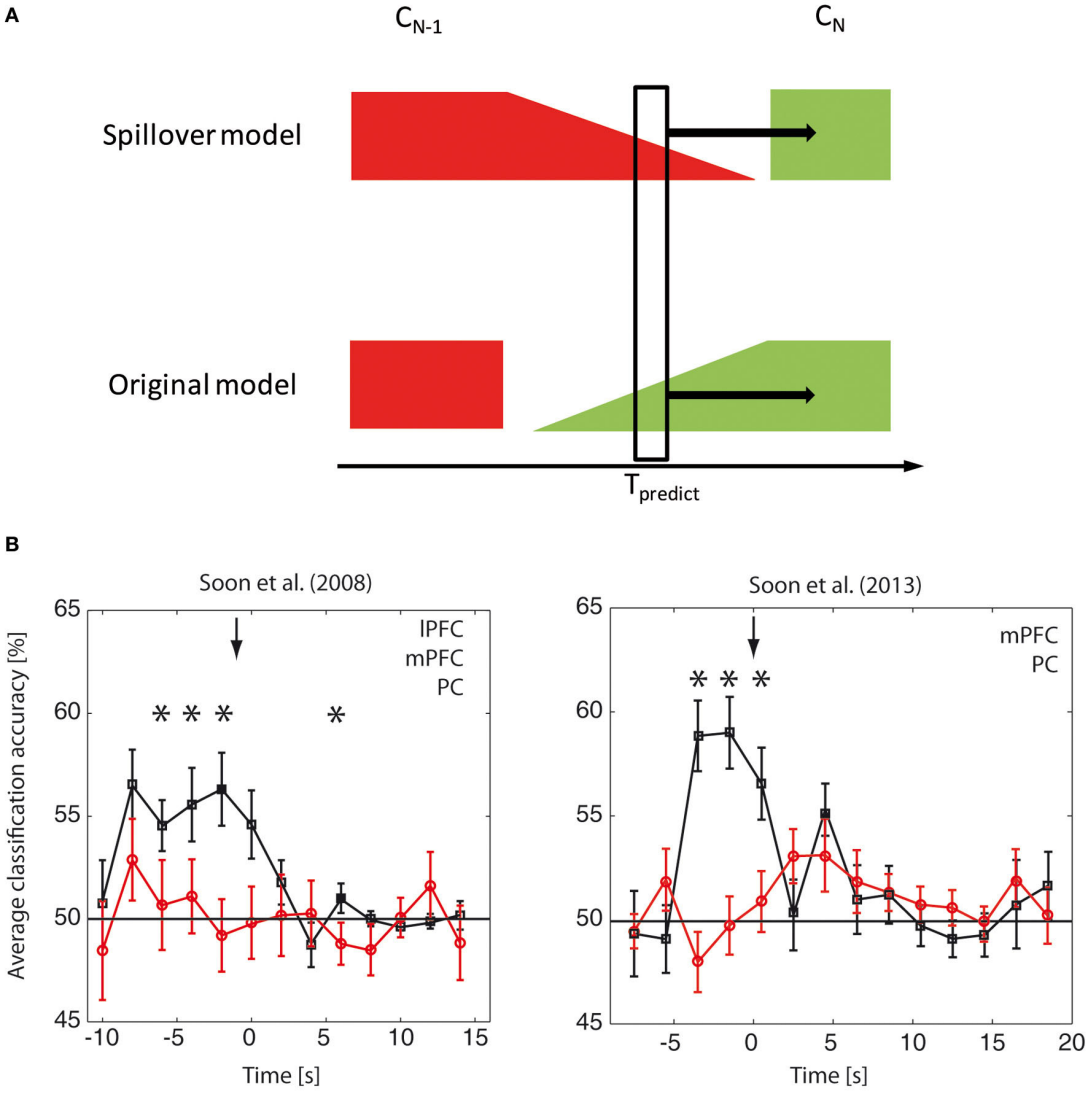
**Choice-predictive brain signals and previous trials**. **(A)** The spillover model assumes that the predictive classification of choice *C*_*N*_ at time T_predict_ is based on some form of residual information lingering from the previous choice *C*_*N* − 1_ (red). The original model assumes that the predictive classification reflects the emergence of choice-related processes (green) that does not directly reflect an explicit representation of the previous trial, even though it presumably evolves in some way from the previous trial based on the causal dynamics of the brain. In the spillover model the prediction of *C*_*N*_ is based alone on signals related to *C*_*N* − 1_ and is due to the fact that the two choices *C*_*N* − 1_ and *C*_*N*_ are correlated. Please note that if this were the case then the signal recorded at T_predict_ should contain substantially more information about *C*_*N* − 1_ than about *C*_*N*_. So if the spillover model is true, it should be possible to decode the previous trial *C*_*N* − 1_ considerably better than the current trial *C*_*N*_. In contrast, if the original model is true, then a shift in labels by trial would largely abolish all predictive signals. **(B)** Reanalysis of choice-predictive brain signals with labels shifted by one trial. For this reanalysis of the original data (left: Soon et al., 2008; right: Soon et al., 2013) we shifted the trial labels by one trial, thus investigating whether a shifted model reflecting a spillover from the previous trial provided a better account for our brain signals. The figures here show data for the shifted analysis in red and for the original analysis in black. The data are collapsed across the three significant clusters lateral prefrontal cortex (lPFC), medial prefrontal cortex (mPFC) and precuneus (PC) for Soon et al. (2008) and collapsed across the two significant clusters medial prefrontal cortex (mPFC) and precuneus (PC) for Soon et al. (2013). Please note that the choice-predictive signals for the original analysis were significant for each region of interest (ROI) individually. For the label-shifted reanalysis they were not-significant at any ROI, thus suggesting that the shifted model does not provide a good account for our data. The collapsing across ROIs in this figure was done in order to increase the statistical power for additionally testing for a *difference* between the original and the shifted analysis. This was necessary due to the fact that the original analysis was tested against a fixed (i.e., “noise-free”) parameter, whereas the statistical power for testing for a difference between the original and shifted analyses is affected by the noise in the shifted classification. Please also note that the baseline accuracies apparent here (and in the original studies) show that the default accuracy is 50%, as expected for two alternative choices. For this reason we did not perform additional permutation tests. (^*^p < 0.05).

The key issue here is that the sole numbers of the trial-by-trial contingencies and the brain-based decoding accuracies alone do not allow us to tell whether the spillover model is true. The only way to decide this issue is to *directly test* whether the spillover model provides a better account of our data. This can be done by applying the same classification analysis as in the original study, but shifted by one trial, i.e., to determine whether neural activity predicting the current trial still encodes the previous decision. If the spillover model is true, the classification accuracy should increase when shifting the labels by one trial. We performed this analysis (see Figure 1B) for both original studies. We found no decodable choice information using the shifted model. In contrast the information from our original model was significant in the seconds leading up to the decision and it was significantly higher than that of the shifted model. Thus, our original model provides the better account of the data and suggests that the choice-predictive signals are not based on a spill-over between trials.

Finally, we would like to point out that we obviously believe that there *has to be* some form of relationship between the brain signals of the two neighboring trials. It would be untenable to assume that choices come out of the blue by some break in the causal flow of events in the brain, rather than by a causal dynamical process that links the previous trial to the current trial. The issue is whether the choice-predictive signals we find are some emergent property of the brain dynamics in the inter-trial period, or whether they reflect a direct and explicit representation of the previous trial, for which we find no evidence in our data.

## Conflict of interest statement

The authors declare that the research was conducted in the absence of any commercial or financial relationships that could be construed as a potential conflict of interest.
